# Seroprevalence of *Coxiella burnetii* antibodies and chronic Q fever among post-mortal and living donors of tissues and cells from 2010 to 2015 in the Netherlands

**DOI:** 10.2807/1560-7917.ES.2018.23.9.17-00384

**Published:** 2018-03-01

**Authors:** Sonja E van Roeden, Eleonoor W Holsboer, Jan Jelrik Oosterheert, Jorge P van Kats, Jacqueline van Beckhoven, Boris M Hogema, Marja J van Wijk

**Affiliations:** 1 Department of Internal Medicine and Infectious Diseases, University Medical Centre Utrecht, Utrecht University, Utrecht, The Netherlands; 2 Dutch Transplant Foundation, Leiden, The Netherlands; 3 BISLIFE Foundation, Leiden (previously Sanquin Bone Bank, Nijmegen), The Netherlands; 4 Sanquin Cord Blood Bank, Leiden, The Netherlands; 5 Sanquin Diagnostic Services, Amsterdam, The Netherlands; 6 Sanquin Plasma Products, Amsterdam (previously BISLIFE Foundation, Leiden), The Netherlands

**Keywords:** Coxiella, Q fever, screening, transplant, tissue donation

## Abstract

After a large Q fever outbreak in the Netherlands in the period from 2007 to 2010, the risk of Q fever transmission through tissue and cell transplantation from undiagnosed chronic Q fever cases became a potential issue. **Aim:** We aimed to evaluate the risk of Q fever transmission through tissue and cell transplantation. **Methods:** We performed a retrospective observational cohort study among 15,133 Dutch donors of tissues and stem cells from 2010 to 2015 to assess seroprevalence of *Coxiella burnetii* antibodies, to identify factors associated with presence of *C. burnetii* antibodies, and to assess the proportion of undiagnosed chronic Q fever cases. **Results:** The study population consisted of 9,478 (63%) femoral head donors, 5,090 (34%) post-mortal tissue donors and 565 (4%) cord blood donors. Seroprevalence of *C. burnetii* antibodies gradually decreased after the outbreak, from 2.1% in 2010 to 1.4% in 2015, with a significant trend in time (p < 0.001). Of 301 seropositive donors, seven (2.3%) were newly detected with chronic Q fever (0.05% of all screened donors). **Conclusion:** This study shows that seroprevalence of *C. burnetii* antibodies among donors of tissues and cells in the Netherlands after 2014 was similar to pre-outbreak levels in the general population. The proportion of newly detected chronic Q fever patients among donors of tissues and cells was smaller than 0.1%. This study may prompt discussion on when to terminate the screening programme for chronic Q fever in donors of tissues and cells in the Netherlands.

## Introduction

Q fever is a zoonosis caused by the intracellular bacterium *Coxiella burnetii*, with a reservoir in a wide range of domesticated and wild animals. The bacterium can be spread via airborne transmission. The Netherlands faced the largest Q fever outbreak ever recorded from 2007 until 2010, resulting in over 4,000 reported and 40,000 estimated infected people. Affected areas were mainly located in the south and east of the Netherlands around infected goat farms [1,2].

Infected animals may be asymptomatic or can show symptoms such as infertility, stillbirth, abortion, endometritis or mastitis [3]. In most human cases (ca 60%), primary infection with *C. burnetii* elapses without symptoms. The remaining proportion of patients develops influenza-like symptoms or more serious conditions such as pneumonia or hepatitis. After acute infection, 1–5% of patients develop chronic infection [3]. The main manifestations of chronic Q fever are endocarditis and vascular infections such as infected aneurysms or vascular grafts [4-6]. Patients most at risk of chronic Q fever are those with underlying valvulopathy, prior valve surgery, aneurysms or vascular prosthesis, or immunocompromised individuals [7-10]. Since the Dutch Q fever outbreak, data from all chronic Q fever patients have been collected systematically for research purposes in the Dutch national chronic Q fever database.

Transmission of *C. burnetii* through tissue transplantation has not been described in literature. However, single cases of likely transmission through blood transfusion and possible transmission through bone marrow transplantation to an immunocompromised recipient have been reported [11,12]. Furthermore, transmission through transplantation (liver, thymus and lymph nodes) in animals has been observed [13]. After the acute phase, bacteria are usually not detectable in the blood, but they can persist in monocytes, bone marrow, spleen, prostate and liver [14]. An assessment showed that a potential risk of transmitting *C. burnetii* lies with transplantation of some tissues such as heart valves, musculoskeletal tissues and skin, although processing techniques may reduce the risk significantly [15].

Since the outbreak of Q fever ended, incidence of acute Q fever in the Netherlands has been low [2]. During a known infection (either acute or chronic), patients are excluded as potential tissue or cell donors in the Netherlands. Therefore, the risk of *C. burnetii* transmission through transplantation of tissues or cells originates mainly from patients with undiagnosed chronic Q fever. In order to reduce the risk of transmission of *C. burnetii* through transplantation and as advised by the Dutch Health Council, testing of all centrally tested donors of tissues and cells was started in 2010 in the Netherlands [16].

The aim of the current study was to assess the seroprevalence of *C. burnetii* antibodies, identify factors associated with the presence of *C. burnetii* antibodies in donors, and to determine the proportion of patients with a serological profile indicative of chronic Q fever among Dutch donors of tissues and cells after the Q fever outbreak in the Netherlands. The proportion of Dutch donors of tissues and cells with chronic Q fever newly detected during this study was compared with the prevalence of known chronic Q fever on a national level. The rationale for this observational cohort study was to generate epidemiological data that may be used in decision-making with regard to continuation of the screening for *C. burnetii* antibodies in donors of tissues and cells.

## Methods

We performed a retrospective observational cohort study among Dutch donors of tissues and cells (post-mortal donors, cord blood donors, bone and cartilage donors) between 2010 and 2015. Data on the prevalence of chronic Q fever in the Netherlands were retrieved from the Dutch national chronic Q fever database.

### Patient selection and data collection for donors

The study included all post-mortal tissue donors who had at least one tissue approved at initial assessment, living donors of femoral heads of Sanquin Bone bank (located in the east of the Netherlands), living donors of femoral heads of BISLIFE (located in the west of the Netherlands) and umbilical cord blood donors from the Netherlands. Besides femoral head donors, cartilage and skullcap donors were included among donors from Sanquin Bone bank. We report the numbers of cartilage and skullcap donors but because their number was very limited, the entire group of bone and cartilage donors was then further referred to as femoral head donors in this manuscript. The donors from these tissue and cell banks represented all post-mortal donors and all cord blood donors in the Netherlands. Bone and cartilage donors were near-complete because a very limited number of bone and cartilage donors are managed and tested in local hospitals. Donors of semen or bone marrow were not included in this study. 

From one donor, multiple types of tissue can be retrieved and each type of tissue can result in multiple transplants for different recipients. As a donor is tested once, in this study, we refer to the number of donors, not the number of derived transplants. The year of screening was the year of initial donation. Tissues can be stored from a month (cornea) up to 5 years (skin, heart valves and musculoskeletal tissues) after retrieval, which may delay the use of tissues. 

No approval of a medical ethical committee was obtained or considered necessary according to Dutch regulations. Screening of post-mortal tissue donors started on 1 November 2010. From June 2012, testing of solo cornea donors was abandoned as the risk of transmission of *C. burnetii* through cornea transplantation was considered very low. Testing of BISLIFE femoral head donors started in June 2012, followed by testing of Sanquin femoral head donors and umbilical cord blood donors from August 2012. All available screening data from 1 November 2010 until 1 January 2016 were included. Donor characteristics, such as age, sex, donated tissues and place of residence were recorded. For all donors who tested positive for *C. burnetii* antibodies and had IgG phase 1 titres ≥ 1:1,024, additional information regarding clinical condition was gathered from hospital medical records, general practitioners, next of kin, autopsy results (if autopsy had been done) and results of histological examination of remnant hearts (if heart valve donation had been performed). The cut-off value of IgG phase 1 titres ≥ 1:1,024 was based upon the diagnostic criteria for chronic Q fever where this titre is indicative of proven, probable or possible chronic Q fever according to the Dutch chronic Q fever consensus group criteria [6]. Besides a phase 1 IgG titre of ≥ 1:1,024, proven chronic Q fever required a definite endocarditis according to the Duke criteria, a positive PCR for *C. burnetii* on tissue, serum or plasma, or a vascular infection diagnosed with imaging studies [17]. Patients with probable chronic Q fever were those who did not meet the criteria for proven chronic Q fever but had a risk factor for chronic Q fever, symptoms consistent with a chronic infection or a focus other than endocarditis or vascular infection. Patients with possible chronic Q fever were those who only had serological evidence of the disease (i.e. a phase 1 IgG titre of ≥ 1:1,024), without fulfilling the definitions of a proven or probable chronic Q fever.

### Patient selection and data collection for chronic Q fever patients

Data on chronic Q fever patients in the Netherlands were retrieved from the Dutch national chronic Q fever database (hosted by the University Medical Centre Utrecht). This database stores clinical, microbiological and radiological data of all known proven, probable and possible chronic Q fever patients older than 18 years in the Netherlands, defined as formulated by the Dutch chronic Q fever consensus group [6]. Registration started in February 2011, and the last update ended in May 2016; 35 hospitals in the Netherlands contributed to the initiative (Supplement 1). Design of this database was approved by the Medical Ethical Committee of the University Medical Centre in Utrecht. Clinicians identified patients based on a positive PCR on serum or tissue and/or a *C. burnetii* phase 1 IgG antibody titre of ≥ 1:1,024. Patients with a serological profile and clinical condition matching acute Q fever were excluded. Details on design and content of this database were described in detail in a previous publication [4]. For calculation of cumulative incidence of chronic Q fever, data on sex and age in the general population were retrieved from the Central Bureau for Statistics [18].

### Definitions

Seropositivity was defined as presence of phase 2 IgG antibodies against *C. burnetii* at a titre ≥ 1:32. Seropositivity without fulfilling the definitions of chronic Q fever indicated a past Q fever infection (symptomatic or asymptomatic). Residency in a high-risk geographical area was defined as living in a four-digit postal code area where at least one Q fever patient had been reported in the 3 months preceding donation, living within a 5 km radius of a farm where *C. burnetii* had been detected in the bulk tank milk at the time of donation, or living in a three-digit postal code area where the Q fever incidence was higher than 20 per 100,000 inhabitants in any of the years 2007 to 2010. Ca 15% of the Dutch population live in an area where 87% of the Q fever cases were reported. The data on Q fever incidence were obtained from the Dutch National Institute for Public Health and the Environment. The data on bulk tank milk-positive farms were obtained from the Dutch Food and Consumer Product Safety Authority website [19]. The 5 km radius from infected farms to the residence of each donor was determined by measuring the distance between both postal codes.

### Laboratory detection of antibodies against *C. burnetii* or of *C. burnetii* DNA 

Serum samples of living donors obtained at the time of donation, or for post-mortal donors within 24 hours after circulation stop, were screened for phase 2 IgG antibodies against *C. burnetii* using the CE-marked Serion enzyme immunoassay (EIA) (Serion, Clindia Benelux, Leusden, the Netherlands). The cut-off values for EIA (borderline) positivity were determined according to the manufacturer’s instructions. Borderline reactive samples were considered positive. Confirmation of positive samples was performed by indirect fluorescent-antibody assay (IFA) for phase 1 and 2 IgG antibodies against *C*. *burnetii* (Focus Diagnostics, Cypress, United States (US)) with dilution on a binary scale, using a cut-off for positivity of 1:32. Laboratory testing of antibodies against *C. burnetii* for chronic Q fever patients from the Dutch national chronic Q fever database consisted of an IFA for phase 1 and 2 IgG against *C. burnetii* on plasma or serum (Focus Diagnostics, Inc., Cypress, US or Fuller Diagnostics, LLC., Anchorage, US). Titration of antibodies was carried out at different hospital sites with dilutions on a binary scale, with a cut-off of 1:32. Moreover, data on PCR for detection of *C. burnetii* DNA on serum or plasma or tissue were collected (NucliSENS easyMAG; bioMérieux, Marcy l'Etoile, France) [4]. 

### Data analysis

All data on donors were stored and analysed in SPSS Statistics 23.0 (IBM SPSS Inc., Chicago, US). Data from the Dutch national chronic Q fever database were stored in Microsoft Access 2010, and exported via R (version i384, 3.1.1) to SPSS Statistics 21.0 for analysis. The numbers of false-positive tissue samples of post-mortal and living donors were compared by means of a chi-squared test. To compare the mean age of seropositive and seronegative donors, an independent samples t-test was used. To identify factors associated with seropositivity, a binomial logistic regression (non-stepwise) was performed, with seropositivity as dichotomous outcome variable. The seroprevalence in time was studied with a Pearson correlation test. The number of newly detected chronic Q fever cases and prevalence of chronic Q fever in the population was not tested formally: numbers of patients were described. We considered p values ≤ 0.05 as statistically significant. Cumulative incidences were calculated for the population per 1,000,000 inhabitants.

## Results

### Donor characteristics

In total, 15,133 donors of tissues and cells were tested for presence of *C. burnetii* antibodies: 10,043 (66.4%) were living donors and 5,090 (33.6%) were post-mortal donors of whom at least one tissue was approved at initial assessment. Among living donors, 9,478 (94.4%) were femoral head donors (3,818 from the bone bank in the east and 5,660 from the bone bank in the west of the Netherlands) and 565 (5.6%) were cord blood donors. Of post-mortal tissue donors, 4,413 (86.7%) donated corneas, 2,535 (49.8%) donated skin, 993 (19.5%) donated cardiovascular tissues and 631 (12.4%) donated musculoskeletal tissues, with multiple tissue types donated by single donors. Donor characteristics are presented in Table 1.

**Table 1:** Sex, age and area of residency for all screened donors of tissues and cells, the Netherlands, 2010–2015 (n = 15,133)

Variable		Donors		Femoral head donors		Cord blood donors		Post-mortal donors	
		n	%^a^	n	%^a^	n	%^a^	n	%^a^
Number of patients		15,133	100	9,478	63	565	4	5,090	34
Sex	Male	7,122	47	3,829	40	NA		3,293	65
	Female	8,011	53	5,649	60	565	100	1,797	35
Mean age in years ± standard deviation		65 ± 14		68 ± 11		32 ± 5		64 ± 13	
Age group (in years)	0–10	27	<1	8^b^	<1	NA		19	<1
	11–20	88	1	46^c^	<1	1	<1	41	1
	21–30	329	2	46	<1	218	39	65	1
	31–40	499	3	51	1	329	58	119	2
	41–50	866	6	404	4	17	3	445	9
	51–60	2,404	16	1,422	15	NA		982	19
	61–70	5,022	33	3,502	37	NA		1,520	30
	71–80	4,685	31	3,071	32	NA		1,614	32
	81–90	1,202	8	917	10	NA		285	6
	91–100	11	<1	11	<1	NA		NA	
Residency in high-risk area	Yes	1,710	11	993^d^	10	33	6	684	13
	No	13,216	87	8,467	89	530	94	4,219	83
	**Unknown**	**207 **	1	**18 **	<1	**2 **	<1	**187 **	4

NA: not applicable.

^a^ Column percentages, except for the first row which shows row percentages.

^b^ Including eight skull-cap donors.

^c^ Including 16 skull-cap donors and five cartilage donors.

^d^ For the bank processing femoral heads in the eastern part of the Netherlands, 21.7% of donors lived in a high-risk area. For the bank processing femoral heads in the western part of the Netherlands, 3.0% of donors lived in a high-risk area (cord blood and post mortal donors were processed centrally).

### Seroprevalence of *C. burnetii* antibodies and characteristics associated with seropositivity

Of 15,133 tested donors, 384 (2.5%) had a positive screening test (EIA); 209 positive and 175 borderline positive. For 353 positive samples (91.9%), a confirmation test (IFA) was done: 83 (23.5%) were negative and were thus considered false positives. For 31 donors with a positive screening test, no confirmation test results were available because the available plasma was not sufficient: these were considered to be true positive. Thus, 301 (2.0%) of all tested donors were considered to be seropositive. Samples of post-mortal donors were significantly more often false positive (n = 46; 0.9%) than tissues of living donors (n = 37; 0.4%; p < 0.001). Mean age did not differ between seropositive (66.4; standard deviation (SD) = 11.4) and seronegative donors (65.3; SD = 13.5; p = 0.14). Seroprevalence of *C. burnetii* antibodies was highest among male donors, donors aged 41–50 years, donors residing in a high-risk area and post-mortal donors. Male sex, residency in a high-risk area and post-mortal donations were independently associated with seroprevalence of *C. burnetii* antibodies in multivariable analysis (Table 2).

**Table 2:** Proportion of donors with antibodies against *Coxiella burnetii* per subgroup and characteristics associated with positive serology, the Netherlands, 2010–2015 (n = 15,133)

Variable		Proportion of seropositive patients in %	OR for seropositivity^a^ (95% CI)	p value for OR
Overall		2.0	NA	NA
Sex	Male	2.7	1.79 (1.40 – 2.29)	p < 0.001
	Female	1.3	Ref	
Age group (in years)	0–10	0	1.08 (0.98 – 1.18)^b^	p = 0.11
	11–20	1.1		
	21–30	0.9		
	31–40	0.8		
	41–50	2.3		
	51–60	2.0		
	61–70	2.1		
	71–80	2.1		
	81–90	1.7		
	91–100	0		
Donor type	Post-mortal	2.8	1.56 (1.23 - 2.00)	p < 0.001
	Living	1.6	Ref	
Residency in high-risk area	Yes	3.7	1.99 (1.50 – 2.64)	p < 0.001
	**No**	**1.8**	**Ref**	

CI: confidence interval; NA: not applicable; OR: odds ratio; Ref: reference category for the variable.

^a^ Seropositivity in individual as dichotomous (yes/no) outcome.

^b^ OR per 10 years increase of age. When only evaluating donors screened after August 2012 (start date for screening of all tissue types), the estimates remained unchanged.

**Figure 1:**
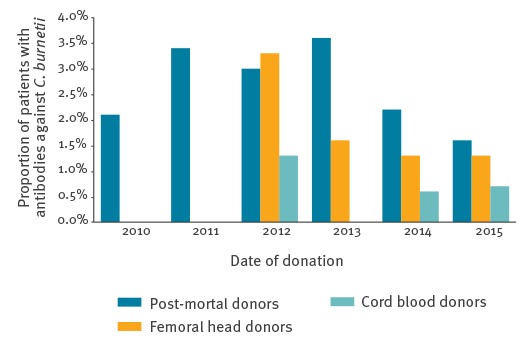
Seroprevalence of antibodies against *Coxiella burnetii* in post-mortal tissue donors, living femoral head donors and cord blood donors per year, the Netherlands, 2010–2015 (n = 15,133)

Among living donors, seroprevalence of *C. burnetii* antibodies was lowest in cord blood donors (3/565; 0.5%). Of the femoral head donors from the western part of the Netherlands, 1.3% (75/5,660) were seropositive. Of the femoral head donors from the eastern part of the Netherlands, 2.1% (79/3,818) were seropositive. Seroprevalence of *C. burnetii* antibodies among all donors gradually decreased in the years after the outbreak from 2.1% in 2010 (5/237), 3.4% in 2011 (53/1,580), 3.1% in 2012 (68/2,228), 1.9% in 2013 (77/4,072), 1.4% in 2014 (59/4,176) and to 1.4% in 2015 (39/2,840), with a significant trend in time (Pearson correlation: r = −0.46; p < 0.001), see Figure 1.

Screening of femoral head donors and cord blood donors started in 2012.

### Chronic Q fever among donors and in the general population

Among all seropositive donors (n = 301), seven patients (2.3%) were newly detected with chronic Q fever during the screening for this study. This corresponds to a proportion of 0.05% people with chronic Q fever among the total of 15,133 donors. In the population in the Netherlands, 443 chronic Q fever patients were identified between January 2007 and May 2016. The cumulative incidence of chronic Q fever in the general population was low: the highest annual incidences were 11.0 per 1,000,000 for men and 5.7 per 1,000,000 for women in 2010, decreasing to 1.7 per 1,000,000 for men and less than 0.5 cases per 1,000,000 for women in 2015. The majority of chronic Q fever patients, both donors and non-donors, were men (74% of the total; n = 331). Furthermore, 75% of chronic Q fever patients (non-donor; n = 333) lived in high-risk areas, whereas none of the donors with chronic Q fever lived in a high-risk area (Table 3). 

**Table 3:** Comparative presentation of donors newly detected with chronic Q fever in this study (n = 7) and chronic Q fever patients in the general population (n = 443), the Netherlands, 2010–2015

Variable	Donors with chronic Q fever	Chronic Q fever patients in the general population
Total number	7	443
Male sex	5 (71%)	326 (74%)
Mean age (years) ± standard deviation	70.3 ± 10	65.8 ± 14
Residency in high-risk area	0	333 (75%)

Donors with newly detected chronic Q fever are not included in column of chronic Q fever patients.

Of screened donors with chronic Q fever, four were post-mortal tissue donors and three were living femoral head donors. In one post-mortal donor with newly diagnosed chronic Q fever, the donated cornea and skin were tested by PCR for presence of *C. burnetii*: both samples were negative. For two other post-mortal donors, histopathological data were available: no signs of endocarditis were present in one donor on examination of the heart after heart valve donation and the other donor had an aortic aneurysm with oesophageal fistula on autopsy. In the fourth donor, no additional evaluation was performed. Two of these living femoral head donors were shown to be PCR-negative for *C. burnetii* on blood (shortly after donation). In the third living femoral head donor, clinically relevant infection was confirmed and chronic Q fever treatment was started. Additional data of donors newly diagnosed with chronic Q fever are presented in Table 4.

**Table 4:** Characteristics of donors with chronic Q fever newly detected during screening of donors, the Netherlands, 2010–2015 (n = 7)

Donor type	Sex	Age group (years)	Year	High-risk area	Risk factors for chronic Q fever	Phase 1 IgG titre	Phase 2 IgG titre
Post-mortal	Male	61-70	2011	No	No	1:4,096	1:4,096
Post-mortal	Female	51-60	2012	No	No	1:2,048	1:2,048
Post-mortal	Male	61-70	2012	No	Aortic aneurysm	1:4,096	1:4,096
Post-mortal	Female	71-80	2013	No	Aortic aneurysm	1:4,096	1:4,096
Living	Male	71-80	2013	No	Aortic aneurysm	1:2,048	1:1,024
Living	Male	71-80	2014	No	Aortic aneurysm	1:2,048	1:2,048
Living	Male	71-80	2014	No	No	1:4,096	1:4,096

**Figure 2:**
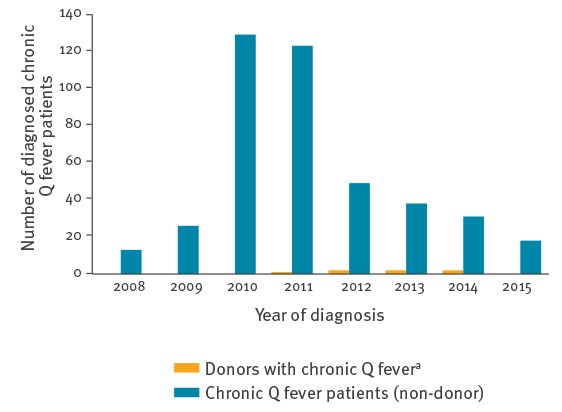
Newly detected chronic Q fever patients in the general population and among donors of tissues and cells, the Netherlands, 2008–2015 (n = 450)

In the general population, the number of newly diagnosed chronic Q fever patients decreased over time (Figure 2). For the majority of chronic Q fever patients, the moment of primary infection was unknown: in 139 patients, time between (serologically confirmed) acute and chronic infection was documented in the Dutch national chronic Q fever database. Mean duration between acute Q fever and chronic Q fever was 61 weeks (SD = 48; range: 0–224 weeks), 91% of all cases of chronic Q fever were diagnosed within two years of acute Q fever. Only four cases of chronic Q fever during pregnancy were reported in the Dutch chronic Q fever database (all with only placenta as focus of infection). In two of these cases, the acute episode was known: the interval between acute infection and chronic infection was 6–8 months. Three cases occurred in 2010 and one case in 2014. No chronic Q fever cases among cord blood donors were detected. 

* The number of donors with chronic Q fever before November 2010 is not known: the screening programme started in November 2010.

## Discussion

Seroprevalence of *C. burnetii* antibodies among donors of tissues and cells in the Netherlands is low, despite the large Q fever outbreak from 2007 to 2010. After the outbreak, seroprevalence in all donors of tissues and cells in the Netherlands peaked in 2011 with 3.4% and decreased significantly over time to 1.4% in 2015. This is similar to the seroprevalence of *C. burnetii* antibodies before the Dutch Q fever outbreak between 2007 and 2010: data from a routine nationwide seroprevalence survey for the National Immunisation Programme in the Netherlands in 2006 and 2007 showed the presence of *C. burnetii* antibodies in 1.5% of people [20]. The decrease in seropositivity among the population of donors of tissues and cells in the Netherlands is comparable with decreasing seroprevalence among blood donors in an outbreak area in the Netherlands, which was 12% in 2009 and 4% in 2012 [21].

Male sex and residency in a high-risk area were associated with seropositivity, which is in line with previous findings from a study among blood donors in high-risk areas and a study describing characteristics of acute Q fever patients [22,23]. Moreover, post-mortal donation was associated with increased seropositivity and increased false-positivity with the screening test (EIA). False positivity may be due to lower sample quality of post-mortal plasma caused by haemolysis, autolysis or bacterial contamination [24]. Therefore, a higher seropositivity in post-mortal samples than in samples of living donors may be explained by the nature and quality of the samples. Cord blood donors had much lower seroprevalence than the other donor groups, which may be explained by the fact that the group only consists of women and that most of the collection sites of the cord blood bank are located in the western part of the Netherlands representing a low-endemic area during the outbreak.

Only seven chronic Q fever patients were detected among screened donors (0.05%). Definite diagnostic classification of chronic Q fever was unknown for these donors, but according to the Dutch chronic Q fever consensus group criteria, four patients would have been diagnosed with at least probable chronic Q fever based on the presence of a risk factor [6]. In none of the donors, blood or tissues were found to be PCR-positive for *C. burnetii*. Remarkably, none of the donors with newly detected chronic Q fever lived in a high-risk area. This may be related to higher awareness among clinicians in high-risk areas and better detection of chronic Q fever patients in routine care (who are excluded from donation), resulting in fewer undiagnosed chronic Q fever patients among donors from high-risk areas.

The results of this study prompt a discussion on whether testing of all donors of high-risk tissues can or should be terminated. The overall number of newly detected chronic Q fever cases was low (< 0.1%). Furthermore, the number of newly detected chronic Q fever patients and seroprevalence in the general population in the Netherlands decreased drastically in the years following the outbreak. The Q fever outbreak subsided more than 6 years ago, and 91% of registered chronic Q fever cases in the Netherlands was diagnosed within 2 years after primary infection. No cases of transmission during or after the outbreak have been reported, despite the fact that extensive and complete screening of all donors of tissues and cells started 2 years after the end of the outbreak. Altogether, it could be argued that screening can be stopped. 

To our knowledge, the Netherlands is the only country in the world in which donors of tissues and cells are screened for Q fever, despite the fact that the disease is endemic in certain areas [25-30]. However, no guidelines are available specifying which level of risk of transmission of pathogens is acceptable for tissue or cell donors: factors such as type of pathogen, consequences of transmission and prevalence of the disease in the population are all factors that should be taken into account. To accurately estimate the possibility of transmission in the absence of screening in the Netherlands, the maximum possible duration between acute infection and chronic infection, the actual prevalence of chronic Q fever in the Netherlands (including undiagnosed cases) and the actual risk of transmission of Q fever after transplanting tissues or cells of an infected donor would have to be known. 

Among patients registered in the Dutch national chronic Q fever database with a known episode of acute Q fever, more than 90% were diagnosed with chronic Q fever within 2 years after primary infection. However, it is questionable if the duration between primary and chronic infection of patients in whom primary infection was recorded is representative for patients in whom primary infection was not recorded. Furthermore, it is possible that incidental cases of chronic Q fever will occur, independently of the epidemic in the Netherlands (a limited number of primary cases are reported every year). Thus, even if the maximum possible interval of developing chronic Q fever after the epidemic has ended, transplantation free of transmission cannot be guaranteed. 

The incidence of both acute and chronic Q fever decreases over time. Since the Q fever outbreak in the Netherlands ended, the number of reported primary Q fever cases has been low with an average of 20 cases per year [3]. The number of newly diagnosed chronic Q fever patients also decreased to 18 cases in 2015. However, from these data it is not possible to accurately estimate the number of undiagnosed chronic Q fever patients in the Netherlands. Moreover, data on the risk of transmission after transplantation of tissues or cells from an infected donor are not available. However, transmission of Q fever after tissue, investigations of femoral head or cord blood donation have not been described in literature.

Altogether, we may conclude that the risk of transmission of Q fever through undiagnosed chronic Q fever patients is very low, but probably not zero. Screening donors only from high-risk areas has been suggested as an alternative to screening all donors, but this seems to be inadequate since none of the newly detected chronic Q fever cases in this study lived in high-risk areas.

To our knowledge this is largest study investigating the outcome of screening donors of tissues and cells for Q fever in a standardised way. Since all post-mortal tissues, all cord blood donations and nearly all bone and cartilage donations are tested centrally, we gathered a near-complete national cohort. Our laboratory protocol was very effective and feasible in practice and may provide guidance for screening programmes globally in endemic or epidemic settings.

Naturally, it can be debated whether these donors were representative of the general Dutch population. However, since *C. burnetii* is highly infectious, the risk of Q fever and consecutive seropositivity is merely dependent on exposure to the pathogen and not on host characteristics [31]. The difference between men and women is believed to be caused by differences in occupational and behavioural exposure (e.g. outdoor smoking) [32]. Since 11.3% of the donors with known residence lived in high-risk areas, similar to the distribution of the general Dutch population, these data are likely to be representative of the general population.

## Conclusion

This study shows that seroprevalence of *C. burnetii* antibodies among donors of tissues and cells in the Netherlands is low (1.4%) and similar to pre-outbreak levels. Furthermore, incidence of chronic Q fever in the general population and the numbers of newly detected chronic Q fever patients among donors of tissues and cells are low (< 0.1%). This study may serve to prompt a discussion of when to terminate screening of donors of tissues and cells for chronic Q fever.
